# The Justifiable Prevention of Conception

**Published:** 1894-06

**Authors:** 


THE JUSTIFIABLE PREVENTION OF CONCEPTION.—The physician not infrequently has to warn against conception in cases where a pregnancy would endanger the life or health of the patient. Pelvic contraction, abdominal and uterine tumors, etc., form such an indication. The advice to abstain from coitus is but seldom followed, and the means usually employed to prevent gestation (mechanical) are objectionable from a hygienic and ethical point of view. Kleinwächter has endeavored to find a remedy which would have none of the aforementioned drawbacks. He prescribes a cacoa butter suppository containing ten per cent. of boracic acid, to be introduced high up into the vagina. These suppositories dissolve in about one hour, and the liberated acid destroys the spermatozoa. Bichloride of mercury in 0.001 gramme doses can also be used, but in that case a vaginal douche has to follow the sexual act. The solvency of the suppository is heightened by adding one grain of oleum olivæ. The author considers this a safe and sure remedy to prevent conception. Therapeutic effects may be combined by the adding of various drugs—for instance, tannin in cases of uterine catarrh.—*Medical Age.*

